# Effects of mineralocorticoid receptor antagonists on coronary microcirculation: a mini-review

**DOI:** 10.3389/fcvm.2026.1855673

**Published:** 2026-10-06

**Authors:** Yu Li, Zhenhong Zhang

**Affiliations:** The First School of Clinical Medicine, Guangdong Medical University, Zhanjiang, China

**Keywords:** cardiovascular diseases (CVD), coronary microcirculation, coronary microvascular dysfunction (CMD), mineralocorticoid receptor (MR), mineralocorticoid receptor antagonists (MRAs)

## Abstract

Mineralocorticoid receptor antagonists (MRAs) serve as a foundational therapies in the management of heart failure, hypertension, and chronic kidney disease. Their primary therapeutic efficacy stems from the competitive antagonism of the mineralocorticoid receptor (MR), which mitigates pathological cascades such as endothelial dysfunction, oxidative stress, chronic inflammation, and perivascular fibrosis. Coronary microvascular dysfunction (CMD)—a pivotal pathological driver across a spectrum of cardiovascular diseases—is defined by impaired vasodilation, heightened microvascular resistance, and diminished coronary flow reserve (CFR). Given that CMD is inextricably linked to dysregulated MR signaling, the restoration of microvascular function represents a key mechanism through which MRAs exert their cardioprotective effects. This review delineates the physiological mechanisms, diagnostic indicators, and generational evolution of MRAs in the context of coronary microcirculation. By synthesizing evidence from animal and clinical studies, this work clarifies the therapeutic utility of MRAs for microvascular protection and provides a comprehensive framework for understanding their pharmacological role in CMD research.

## 1 Introduction

Cardiovascular diseases (CVD) remain the leading cause of global mortality, driven by intricate pathophysiological mechanisms. While traditional therapies have historically focused on macrovascular interventions, the rising clinical recognition of ischemia associated with non-obstructive coronary artery disease (INOCA) has shifted the paradigm toward the coronary microcirculation, which comprises the vast majority of the coronary tree. CMD has emerged as a central clinical entity; it is a primary driver of stable angina and serves as the fundamental pathological substrate for myocardial injury in heart failure with preserved ejection fraction (HFpEF) and diabetic cardiomyopathy. Characterized by structural and functional impairments in pre-arterioles, arterioles, and capillaries, CMD manifests as diminished vasodilatory capacity, heightened microvascular resistance, and reduced CFR. These abnormalities are fundamentally linked to endothelial dysfunction, oxidative stress, chronic inflammation, and adverse microvascular remodeling.

Throughout this pathological process, the overactivation of the MR serves as a central driver of vascular decline (3, 4). The MR is widely expressed across the myocardium, vascular endothelium, and smooth muscle cells, where its aberrant activation triggers a cascade of genomic and non-genomic effects. These pathways directly induce endothelial dysfunction, oxidative stress, chronic inflammation, and interstitial fibrosis, collectively facilitating the onset and progression of CMD (1–3, 5). Given this mechanistic link, the MR represents a highly promising therapeutic target for CMD intervention. Consequently, the clinical utility of MRAs has undergone a significant paradigm shift, transitioning from their traditional role as potassium-sparing diuretics to sophisticated agents for heart failure and hypertension management, with an increasing focus on systemic vascular protection. The potential for MRAs to ameliorate coronary microvascular impairment is rooted in their pleiotropic ability to restore endothelial integrity, neutralize reactive oxygen species, and suppress the pro-fibrotic signaling that compromises microcirculatory health. Ultimately, MRA therapy may offer a novel and potent pharmacological strategy for the targeted treatment of CMD.

This review examines the impact of MRAs on the coronary microcirculation, analyzing pharmacological mechanisms and synthesizing evidence across disease models to establish a theoretical framework for targeted CMD therapy. This work aims to provide a novel perspective for treating CMD.

## 2 Definition of coronary microcirculation

The coronary vasculature forms a sophisticated, continuous network traditionally categorized into four distinct functional segments (6). The epicardial coronary arteries, characterized by internal diameters ranging from 0.5 to 5.0 mm, primarily serve a capacitance function and exert minimal influence on overall blood flow resistance. In contrast, pre-arterioles, with diameters between 100 and 500 μm, are the principal regulators of coronary perfusion pressure and contribute significantly to vascular resistance. Pre-arterioles are further subdivided into proximal pre-arterioles (150–500 μm), which respond primarily to flow-mediated stimuli, and distal pre-arterioles (100–150 μm), which are highly sensitive to pressure fluctuations.

The third segment comprises the arterioles, which possess diameters of less than 100 μm and are uniquely responsive to myocardial metabolite concentrations, thereby regulating the critical balance between myocardial oxygen supply and demand (7). Finally, the capillaries, consisting of a single endothelial cell layer, constitute 90% of the myocardial vascular bed and are responsible for essential nutrient delivery, oxygenation, and metabolic waste exchange (8). Collectively, the pre-arterioles, arterioles, and capillaries define the coronary microcirculation. The significance of this microvascular network is highlighted by the distribution of the pressure gradient from the aorta to the coronary sinus: while the epicardial arteries account for only 10% of the pressure drop, the pre-arterioles and arterioles represent 30% and 40%, respectively, with the remaining 20% occurring within the capillaries and the venous system. Consequently, the coronary microcirculation serves as the primary site for the regulation of both perfusion pressure and total coronary blood flow (9).

## 3 Evaluation indicators of coronary microcirculation

### 3.1 Coronary blood flow (CBF)

CBF represents the volume of blood flowing through the coronary artery system per unit of time, usually expressed in milliliters per minute (mL/min) or milliliters per minute per 100 grams of myocardial tissue (mL/min/100 g). CBF can be measured using either the bolus injection thermodilution method or the continuous thermodilution method. It is a key physiological parameter for maintaining myocardial oxygen supply and energy metabolism, and it is directly related to the normal functioning of the heart.

### 3.2 Coronary flow reserve (CFR)

CFR represents the ratio of CBF or myocardial blood flow (MBF) at near-maximum coronary artery dilation to the corresponding indicators at rest, serving as an overall measure of the reserve function of the entire coronary artery system. CFR can be measured via positron emission tomography (PET), single-photon emission computed tomography (SPECT), cardiac magnetic resonance (CMR), coronary angiography, myocardial contrast echocardiography and radiomicrosphere method (10–12). CFR is influenced by four factors: resting CBF, the cross-sectional area of resistance vessels per unit volume of myocardium, extravascular pressure on the coronary arteries, and coronary perfusion pressure.

### 3.3 Coronary flow velocity reserve (CFVR)

CFVR is calculated using transthoracic echocardiographic CBF imaging to measure the maximum diastolic flow velocity of the subepicardial coronary artery under adenosine/dipyridamole/regadenoson stress relative to rest (13). A measured value ≤ 2.0–2.5 generally indicates impaired coronary microvascular function (14), and clinically CFR <2.0 is recommended as the threshold for assessing microvascular dysfunction (15).

### 3.4 Myocardial blood flow (MBF)

MBF represents the amount of blood flowing through a unit mass of myocardial tissue per unit of time, and is typically expressed in milliliters per minute per gramof myocardium (mL·min⁻¹·g⁻¹). This parameter is a core quantitative indicator for assessing myocardial perfusion status and directly reflects the functional integrity of the myocardial microcirculation system and its oxygen supply capacity. MBF can be measured using PET, SPECT, CMR, or CT perfusion imaging (CTP). Measuring MBF can reveal the blood supply status of the local or overall myocardium under both resting and stress conditions (such as adenosine, dipyridamole, or exercise load). This enables the calculation of the myocardial perfusion reserve (MPR)—the ratio of stress MBF to resting MBF—which is used to identify early microvascular dysfunction or perfusion limitation caused by epicardial coronary artery stenosis.

### 3.5 Myocardial perfusion reserve (MPR)

MPR represents the ratio of MBF at maximum hyperemia to that at rest, used to assess coronary microcirculation function and MBF reserve capacity. MPR can be measured through CMR or computed tomography perfusion imaging. This indicator reflects the ability of the coronary arteries to increase blood flow under physiological or pathological stimulation. It is an important parameter for evaluating myocardial ischemia and microcirculatory dysfunction, as well as a strong marker of the myocardium's ability to adapt its blood supply to an increased myocardial workload.

### 3.6 Index of microvascular resistance (IMR)

IMR is an index proposed for evaluating microvascular function distal to coronary artery stenosis (16). It is defined as the distal pressure (Pd) of the stenotic lesion under coronary hyperemia divided by 1/T (the mean transit time), which represents the ratio of pressure to flow. Pd and T can be measured using a pressure guidewire equipped with a temperature sensor (17). IMR is independent of epicardial vessel function and can specifically evaluate microvascular function distal to a stenotic lesion with relatively good reproducibility (17–19).

### 3.7 Microvascular resistance reserve (MRR)

MRR is an index for evaluating microvascular function, defined as the ratio of microcirculatory resistance at rest to that during hyperemia (20). The calculation formula is expressed as: MRR = (Qmax/Qrest) × (Pa,rest/Pd,hyper). (Pa,rest) represents aortic pressure at rest, (Pd,hyper) represents distal coronary pressure during hyperemia, Qrest and Qmax represent measured blood flow at rest and during hyperemia, respectively. MRR can be obtained through invasive methods such as intracoronary Doppler, continuous thermodilution, or bolus thermodilution measurements. Although the optimal threshold for MRR has yet to be determined, exploratory analyses in patients with angina without obstructive coronary artery disease have shown that an MRR >2.7 effectively rules out CMD, whereas an MRR <2.1 strongly suggests CMVD (20). MRR provides measurement accuracy, good reproducibility, and safety. Furthermore, it remains unaffected by epicardial coronary artery disease, hemodynamic changes, vascular autoregulation, epicardial vascular resistance, myocardial mass, or operator skill, making it highly significant clinically.

### 3.8 Hyperemic microvascular resistance (HMR)

HMR is a parameter described in 2023 (21). This technique uses a coronary Doppler flow-pressure wire to measure the average flow velocity and pressure during maximal hyperemia of the cardiac cycle distal to a stenotic lesion (or in the distal coronary artery if no stenotic lesion is present). The formula is expressed as: HMR = pressure/flow velocity (22). Structurally, a CFR < 2.5 and HMR > 1.7 mmHg·cm⁻¹·s⁻¹are defined as CMD.

### 3.9 Myocardial perfusion reserve Index (MPRI)

MPRI is the core quantitative indicator for non-invasively assessing myocardial microcirculatory function through CMR imaging. It is defined as the ratio of MBF measured under pharmacologically induced hyperemia (usually using adenosine or dipyridamole) to MBF at rest, MPRI = MBFstress/MBFrest. The normal reference value for MPRI is usually ≥2.0. When the MPRI falls below this threshold (e.g., <2.0), it indicates impaired MPR, which often suggests the presence of CMD.

### 3.10 Fractional flow reserve (FFR)

FFR is the core clinical indicator for assessing the physiological significance of epicardial coronary artery stenosis. It is defined as the ratio of the maximum blood flow that a stenotic coronary artery can provide under conditions of maximal hyperemia to the maximum blood flow that the same artery could achieve in an ideal, non-stenotic state. FFR is obtained through synchronous measurements during coronary angiography: after inducing maximal hyperemia via the administration of vasodilators such as adenosine, a pressure wire is used to record the coronary pressure distal to the stenosis (Pd) and the aortic root pressure (Pa), FFR is calculated as the ratio of the two values (FFR = Pd/Pa) (23). In a healthy coronary artery, the FFR value is 1.0, whereas hemodynamically significant stenosis causes the FFR value to decrease.

## 4 Definition of Coronary microvascular dysfunction

CMD is a clinical syndrome characterized by acute or chronic myocardial ischemia secondary to structural and functional abnormalities of the prearterioles, arterioles, and capillaries. Driven by both atherosclerotic and non-atherosclerotic pathways (24), CMVD was formally recognized by the 2017 Chinese Expert Consensus (25) and further refined its 2023 update Chinese Expert Consensus (24), which integrated recent clinical advancements to standardize the diagnostic and therapeutic frameworks for the disease.

The core pathophysiology of CMVD stems from structural or functional impairments within the coronary microcirculation. Key pathogenic drivers—including endothelial dysfunction, chronic inflammation, microvascular vasospasm, and platelet activation—disrupt the delicate balance of vascular tone. These mechanisms culminate in abnormal vasoconstriction and impaired vasodilation, which significantly compromise myocardial perfusion and cellular metabolism, thereby leading to an oxygen supply-demand mismatch (26, 27).

Functional microvascular impairment in CMVD leads to myocardial hypoxia and ischemia, manifesting clinically as angina or overt myocardial injury. Beyond manifesting as acute symptoms, CMVD significantly drives major adverse cardiovascular events (MACE) and progression toward heart failure. Its integral role in the pathogenesis of diabetes and coronary heart disease has positioned the microvasculature as a critical therapeutic target for improving long-term clinical outcomes across multiple metabolic and cardiac conditions.

## 5 Mechanisms of mineralocorticoid receptor action on the coronary microcirculation

MR overactivation is a core pathological driver of CMD, acting through three highly intertwined signaling pathways—oxidative stress, chronic inflammation, and vascular fibrosis—to cause severe impairment of microcirculatory structure and function (2, 28). While MR is classically expressed in renal epithelial cells to regulate fluid-electrolyte balance, it is also widely expressed in non-epithelial cells of the cardiovascular system, including vascular endothelial cells, smooth muscle cells, fibroblasts, and immune cells (3, 29). Under the pathological context of CMD, aberrant MR activation triggers a cascade of reactions that ultimately lead to inadequate myocardial perfusion, which is particularly prominent in patients with HFpEF and INOCA (30, 31).

### 5.1 Oxidative stress: the initiating event in microvascular injury

Oxidative stress is an early and critical molecular event in MR-mediated microvascular dysfunction. Upon MR activation, transcriptional regulatory mechanisms significantly upregulate the expression of subunits of nicotinamide adenine dinucleotide phosphate (NADPH) oxidase, particularly Nox2, Nox4, and p22phox; these enzyme complexes are the primary sources of reactive oxygen species (ROS) in the vascular wall (2, 3, 28, 29). In coronary microvascular endothelial cells, MR-driven ROS bursts directly consume nitric oxide (NO), leading to a sharp decline in NO bioavailability (32). At the same time, MR activation inhibits endothelial nitric oxide synthase (eNOS) activity and upregulates inducible nitric oxide synthase (iNOS) expression, further exacerbating the redox imbalance (29). Because NO is a key mediator for maintaining microvascular relaxation and inhibiting platelet aggregation, its deficiency directly impairs endothelium-dependent vasodilation (33). In addition, high levels of ROS activate protein kinase C (PKC) and mitogen-activated protein kinase (MAPK) signaling pathways, enhancing vascular smooth muscle cell contraction and altering microvascular permeability, thereby disrupting microcirculatory hemodynamic stability (29). This oxidative stress state not only damages the endothelial barrier but also provides a molecular basis for subsequent inflammatory responses through activation of nuclear factor-*κ*B (NF-*κ*B) (34).

### 5.2 Inflammatory cascade: a core mechanism of perimicrovascular injury

MR activation in immune cells and endothelial cells is a central hub driving chronic low-grade inflammation in coronary microvessels. The downstream serum- and glucocorticoid—regulated kinase-1 (SGK1) signaling pathway of MR plays a key role in triggering inflammasome assembly (34). Specifically, MR activation promotes NLRP3 inflammasome assembly and activation, which in turn activates caspase-1, leading to maturation and secretion of interleukin-1*β* (IL-1*β*) (35). Mature IL-1*β* further stimulates the release of pro-inflammatory cytokines such as interleukin-6 (IL-6) and tumor necrosis factor-*α* (TNF-*α*), establishing a persistent inflammatory microenvironment (2). Meanwhile, MR activation upregulates vascular cell adhesion molecule-1 (VCAM-1) and intercellular adhesion molecule-1 (ICAM-1) expression, significantly enhancing leukocyte-endothelial adhesion and transendothelial migration (28). This immune cell infiltration leads to the formation of inflammatory cuffs around microvessels, which not only physically compress the microvascular lumen and restrict blood flow but also disrupt vascular wall integrity through paracrine effects (36). In CMD patients, this sustained inflammatory state is closely associated with myocardial stiffness and diastolic dysfunction, constituting an important component of HFpEF pathophysiology (30).

### 5.3 Fibrotic remodeling: the final common pathway to microcirculatory structural stiffening

Vascular fibrosis is the final common pathway of structural pathology in CMD, where MR promotes excessive extracellular matrix (ECM) deposition through both direct and indirect mechanisms. MR activation significantly upregulates transforming growth factor-*β* (TGF-*β*) expression and enhances Smad2/3 phosphorylation and nuclear translocation, thereby initiating the TGF-*β*/Smad signaling pathway. This pathway stimulates the transformation of vascular fibroblasts into myofibroblasts and markedly increases the synthesis of type I and type III collagen and fibronectin. At the same time, MR activation suppresses matrix metalloproteinase (MMP) activity and upregulates the expression of their tissue inhibitors (TIMPs), leading to impaired ECM degradation. This imbalance between increased synthesis and reduced degradation results in microvascular wall thickening, luminal narrowing, and a marked decrease in vascular compliance. More importantly, MR activation induces endothelial-to-mesenchymal transition (EndMT), causing endothelial cells to lose their original phenotype and acquire fibroblast-like characteristics, directly participating in fibrotic matrix formation (2, 28). This structural remodeling manifests as capillary rarefaction and severe perivascular fibrosis, significantly increasing microvascular resistance and limiting myocardial perfusion capacity when metabolic demand rises (33).

## 6 The effects of mineralocorticoid receptor antagonists on coronary microcirculation

MRAs represent a cornerstone in the management of cardiovascular and renal diseases, evolving from fundamental diuretics into sophisticated therapeutic agents with systemic protective capabilities. These drugs function by selectively antagonizing the MR to disrupt pathological aldosterone signaling pathways. Their clinical utility extends significantly beyond traditional diuresis, as they actively suppress aldosterone overactivation within the kidneys, heart, vasculature, and immune system. By arresting these pathways, MRAs mitigate critical pathological processes—such as chronic inflammation, progressive fibrosis, adverse organ remodeling, and sodium-water retention—thereby providing comprehensive cardiorenal protection. Based on their distinct chemical structures, developmental history, and pharmacological profiles, MRAs are categorized into first- and second-generation steroidal agents, including spironolactone and eplerenone, and third-generation non-steroidal agents, such as finerenone. Theoretically, the ameliorative effect of MRAs on coronary microcirculation may represent a novel mechanism underlying their cardiovascular benefits. To test this hypothesis, we reviewed available evidence from relevant animal experiments and clinical trials.

### 6.1 Effects of mineralocorticoid receptor antagonists on coronary microcirculation in animal models

In a 2005 study by Susic et al. focusing on spontaneously hypertensive rats (SHRs), 22-week-old males were randomized into four experimental cohorts of 10 animals each to evaluate the impact of MR blockade on coronary health. The researchers established a baseline control group alongside three treatment arms receiving either eplerenone (100 mg/kg/day), lisinopril (3 mg/kg/day), or a combination of both agents. Following a 12-week regimen, CFR was quantified using the radioactive microsphere method to assess hemodynamic performance. The findings revealed that eplerenone significantly improved coronary hemodynamics, as evidenced by a marked increase in CFR—an effect that was further amplified in the combination treatment group. Notably, lisinopril monotherapy yielded no significant improvement in coronary reserve, suggesting that eplerenone prevents hypertension-related hemodynamic impairment through a specific mechanism likely involving the restoration of vascular endothelial function (37).

In a 2010 follow-up study investigating the role of the renin-angiotensin-aldosterone system (RAAS) in high-salt-induced cardiovascular injury, Susic et al. subjected 8-week-old male SHRs to a rigorous dietary and pharmacological protocol. While the control group was maintained on a standard 0.6% NaCl diet, the remaining six groups were challenged with an 8% NaCl solution to simulate high-salt-induced damage. These cohorts were then randomized to receive either a placebo, eplerenone (100 mg/kg/day), quinapril (3 mg/kg/day), candesartan (10 mg/kg/day), or combinations of eplerenone with either quinapril or candesartan over an 8-week period. The results indicated that baseline CBF remained remarkably stable, as it was not significantly altered by either the high-salt loading or the various RAAS inhibitors, including eplerenone (38).

In that same work utilizing a high-salt diet (8% NaCl) in young SHRs to exacerbate cardiovascular injury, eplerenone monotherapy demonstrated only modest improvements in CFR and other indices of cardiac and renal damage (32). In contrast, treatment with either the ACEI quinapril or the ARB candesartan, alone or in combination, was significantly more effective in normalizing these parameters. Crucially, the coronary microvascular injury induced by high-salt loading (as indicated by reduced CFR) was primarily driven by angiotensin II rather than by the aldosterone/MR pathway. Therefore, the efficacy of eplerenone in this context was less pronounced than that of ACEIs/ARBs, indicating that the underlying pathophysiology was driven by angiotensin II rather than high salt directly “counteracting” its benefits. This finding does not negate the potential benefits of MRAs in other contexts, but it highlights that the therapeutic efficacy of MRAs is highly dependent on the underlying pathological model and the relative contribution of MR activation to the disease process. It also underscores the need for further studies to clarify the interaction between dietary sodium and the microvascular protective effects of MRAs.

Obstructive sleep apnea (OSA), defined by chronic intermittent hypoxia (IH), serves as a potent independent risk factor for cardiovascular disease. While MR blockade is known to improve outcomes in related conditions (39, 40), its role in preventing coronary vascular dysfunction during OSA-related stress has only recently been elucidated. In a 2024 study, Mohammad Badran et al. exposed male mice to an IH environment for 12 h per day over six consecutive weeks to establish a robust OSA model, with cohorts treated either with or without spironolactone. Following the 6-week intervention, coronary artery blood flow velocity was assessed via a handheld 20 MHz Doppler probe to calculate the CFVR, defined as the ratio of hyperemic peak velocity to baseline peak velocity (H/B). The results demonstrated that spironolactone treatment significantly enhanced CFVR in mice subjected to IH, reaching (3.4 ±0.3, *p* < 0.001). These findings indicate that spironolactone effectively preserves coronary microvascular function in the setting of OSA, likely by mitigating the pathological signaling pathways triggered by chronic intermittent hypoxia (41).

Experimental evidence across models of hypertension and intermittent hypoxia-induced vascular dysfunction confirms that MRAs, such as eplerenone and spironolactone, exert substantial protective effects on the coronary microvasculature. These benefits are primarily manifested through the significant enhancement of CFR or CFVR. The underlying mechanism of this improvement transcends simple systemic blood pressure reduction; rather, it is rooted in the restoration of vascular endothelial function and the mitigation of oxidative stress and inflammatory signaling. However, emerging research indicates (38) that a high-sodium load can biologically mask or attenuate these microcirculatory benefits, suggesting that dietary salt intake is a critical determinant of drug efficacy. Consequently dietary sodium restriction may serve as a **crucial** prerequisite for the therapeutic success of MRA therapy. In clinical practice, the strategic integration of sodium restriction with MRA administration appears to be an important approach to maximizing their cardiorenal protective value (Table 1).

**Table 1:** Animal study of mineralocorticoid receptor antagonists on coronary artery microcirculation.

Study (year)	Group	Animals	Duration	Measuring tools	Measured index	Outcome
Susic D (2005) (37)	Control group: Blank Control Eplerenone group: Eplerenone (100 mg/kg/day) Lisinopril group: Lisinopril (3 mg/kg/day) Combined treatment group: Combined treatment with Eplerenone and Lisinopril	48 spontaneously hypertensive rats	12 weeks	radiomicrosphere method	CFR	Eplerenone group vs. Control group (*P* ＜ 0.05), Combined treatment group vs. Control group (*P* ＜ 0.05)
Susic D (2010) (38)	Group 1: Standard feed containing 0.6% NaCl + Tap water Group 2: Rat feed containing 8% NaCl + Distilled water Group 3: Rat feed containing 8% NaCl + Spironolactone (100 mg/kg/day) Group 4: Rat feed containing 8% NaCl + Quinapril (3 mg/kg/day) Group 5: Rat feed containing 8% NaCl + Candesartan (10 mg/kg/day) Group 6: Rat feed containing 8% NaCl + Spironolactone (100 mg/kg/day) +Quinapril (3 mg/kg/day) Group 7: Rat feed containing 8% NaCl + Eplerenone (100 mg/kg/day)+ Candesartan (10 mg/kg/day)	105 spontaneously hypertensive rats	8 weeks	radiolabeled (^46^Sc) microspheres	CBF	Group 1: 5.3 ± 0.5 (ml/min/g) Group 2: 4.8 ± 0.8 (mL/min/g) Group 3: 5.1 ± 0.5 (mL/min/g) Group 4: 5.0 ± 0.5 (ml/min/g) Group 5: 4.6 ± 0.3 (mL/min/g) Group 6: 4.9 ± 0.4 (mL/min/g) Group 7: 5.0 ± 0.5 (mL/min/g) Coronary hemodynamics at basal conditions was not statistically affected either by salt overload or by various RAAS blockers.
Badran M (2025) (41)	Control group: Mice exposed to intermittent hypoxic conditions without treatment for 6 weeks (*n* = 6) Spironolactone group: Mice exposed to intermittent hypoxic conditions treated with spironolactone (20 mg/kg/day) for 6 weeks (*n* = 5)	11 OSA mice	6 weeks	Doppler probe	CFVR	Spironolactone group vs. Control group (3.4 ± 0.3 vs 2. 6 ± 0.2, *p* < 0.001)

CFR, Coronary Flow Reserve; CBF, Coronary Blood Flow; CFVR, Coronary Flow Velocity Reserve.

### 6.2 Effects of mineralocorticoid receptor antagonists on coronary microcirculation in patients

In a 2007 randomized, double-blind, crossover trial, Joffe et al. (42) investigated whether the addition of an MRA could further enhance the coronary microcirculation in diabetic patients already optimized on angiotensin-converting enzyme inhibitor (ACEI) therapy. The study enrolled 16 patients with diabetes and proteinuria—all maintained on enalapril (20 mg/day)— who were randomized to receive either eplerenone (50 mg/day) or hydrochlorothiazide (12.5 mg/day) for 6-week intervals in a randomized sequence. To assess microvascular health, the researchers utilized CMR to measure adenosine-stimulated MPR. While baseline MPR values were comparable between the two cohorts, post-treatment results revealed that eplerenone achieved a significantly higher MPR than hydrochlorothiazide, with a median of 1.57 versus 1.30 (*P* = 0.03). These findings demonstrate that even in the presence of existing RAAS blockade, short-term treatment with eplerenone provides superior improvement in coronary circulatory function for diabetic patients with albuminuria compared to standard diuretic therapy.

In a 2015 double-blind, randomized, controlled trial, Garg et al. (43) evaluated whether MR blockade could enhance CFR specifically in patients with type 2 diabetes mellitus (T2DM). The study randomized 64 diabetic patients—all maintained on a stable dose of enalapril (20 mg/day)—to receive either spironolactone (25 mg/day), hydrochlorothiazide (12.5 mg/day), or a matching placebo for a duration of six months. To quantify microvascular function, the investigators utilized cardiac PET to measure CFR at baseline and at the study's conclusion. The data revealed that spironolactone treatment led to a significant increase in CFR compared to the hydrochlorothiazide cohort (0.33 vs. −0.10, *P* = 0.04). The researchers concluded that the addition of spironolactone to standard therapy, including an ACEI, effectively improves coronary microvascular function in well-controlled T2DM patients, even in the absence of clinically manifest ischemic heart disease.

In a 2023 double-blind, randomized, placebo-controlled trial, Srinivasa et al. (44) investigated the capacity of MR blockade to enhance myocardial perfusion in people living with HIV (PWH) on stable antiretroviral therapy for over 12 months. Utilizing a permuted block randomization algorithm, 40 participants were assigned in a 1:1 ratio to receive either eplerenone or a matching placebo for one year. The eplerenone regimen commenced at 50 mg/day for one week before escalating to a maintenance dose of 50 mg twice daily. While a significant portion of the cohort exhibited impaired CFR or stress myocardial blood flow (sMBF) at baseline, eplerenone treatment in this specific subgroup resulted in a significant improvement in CFR compared to the placebo group (0.28 vs. −0.05, *P* = 0.04). Conversely, no such benefit was observed in participants with normal baseline flow parameters (*P* = 0.99). These results suggest that the therapeutic efficacy of eplerenone on the coronary microcirculation is selectively optimized in patients with pre-existing myocardial perfusion impairment.

Endothelial dysfunction is highly prevalent among patients with non-obstructive coronary artery disease and is strongly associated with adverse clinical outcomes (45). Although inhibiting angiotensin II is a standard strategy to improve endothelial health, the phenomenon of “aldosterone breakthrough” (46) during chronic treatment often limits the efficacy of this approach. To determine if supplemental MR blockade could overcome this limitation, Bavry et al. conducted a multicenter, double-blind, randomized trial involving women presenting with ischemic symptoms and coronary endothelial dysfunction in the absence of significant obstructive lesions. The study randomized 70 participants, all of whom were already receiving an ACEI or an angiotensin II receptor blocker (ARB), to receive either eplerenone—titrated from 25 mg/day to 50 mg/day—or a matching placebo. Of these, 19 patients in the eplerenone group and 22 in the placebo group completed the 16-week protocol and underwent follow-up cardiac catheterization. The final analysis revealed that the change in CFR was not statistically significant between the two groups. Consequently, the study concluded that adding aldosterone blockade to existing angiotensin II inhibition does not improve CFR in women with non-obstructive coronary artery disease who present with symptomatic ischemia (47).

Clinical evidence indicates that MRAs effectively enhance coronary circulatory function in patients with diabetes and HIV, particularly among those with pre-existing myocardial perfusion impairment. In these populations, MRAs demonstrate a significant additive benefit when integrated with standard ACEI therapy. Conversely, the impact of MRAs on the coronary microcirculation in women with non-obstructive coronary artery disease remains unconfirmed, as clinical trials in this specific demographic have yielded neutral results. Ultimately, the therapeutic application of MRAs for coronary microvascular protection requires a nuanced, individualized approach that accounts for the patient's specific underlying clinical condition and baseline perfusion status (Table 2).

**Table 2:** Clinical study of mineralocorticoid receptor antagonists on coronary artery microcirculation.

Study (year)	Group	Patients	Duration	Measuring tools	Measured index	Outcome
Joffe HV 2007 (42)	Eplerenone group:Eplerenone (50 mg/day) + placebo pill daily HCTZ group:Hydrochlorothiazide (12.5 mg/day) + potassium chloride 10 mEq daily	16 diabetic patients	6 weeks	CMR	MPR	Eplerenone group vs. Hydrochlorothiazide group (1.57 vs. 1.30; *P* = 0.03)
Bavry AA 2014 (47)	Eplerenone group: Eplerenone (25 mg/day, taken continuously for 4 weeks, then changed to 50 mg/day, taken continuously for 12 weeks) *n* = 19 Control group: Matching placebo (1 tablet/day, used continuously for 4 weeks, then gradually increase the dose, used continuously for 12 weeks) *n* = 22	41 women with coronary endothelial dysfunction treated with ACEI or ARB	16 weeks	6F guiding catheter	CFR	Eplerenone group vs. control group (2.64 ± 0.62 vs 2.79 ± 0.52; *P* = 0.46)
Garg R 2015 (43)	Spironolactone group: Spironolactone (25 mg/day) Hydrochlorothiazide group: Hydrochlorothiazide (12.5 mg/day) Placebo group: Matched Placebo	64 patients with T2DM receiving enalapril 20 mg/day	6 months	PET	CFR change value	Spironolactone Group vs. Hydrochlorothiazide Group (0.33 ± 0.83 vs −0.1 ± 0.65, *P* = 0.04)
Srinivasa S 2023 (44)	Eplerenone group: The starting dose of eplerenone is 50 mg/day for 1 week, then increased to 50 mg per time, twice a day, for 12 months (*n* = 20) Placebo group: Matched Placebo (*n* = 20)	40 people with HIV (PWH)	12 months	PET	CFR change value	Overall population: Eplerenone group vs. Placebo group (0.01 vs - 0.07; *P* = 0.72) Among the CFR-impaired population: eplerenone group vs. placebo group (0.28 vs - 0.05; *P* = 0.04) CFR in the normal population: Eplerenone group vs. placebo group (no statistical significance, *P* = 0.99)

CMR, Cardiovascular Magnetic Resonance; MPR, Myocardial Perfusion Reserve; CFR, Coronary Flow Reserve; PET, Positron Emission Tomography.

## 7 Conclusion

Evidence from animal models and clinical studies indicates that MRAs, such as spironolactone and eplerenone, possess significant potential to ameliorate CMD. By improving endothelial function, reducing oxidative stress, and inhibiting inflammatory and fibrotic pathways, these agents can substantially enhance key microvascular indicators, including coronary flow reserve (CFR), coronary flow velocity reserve (CFVR), and myocardial perfusion reserve (MPR). However, the therapeutic impact of MRAs varies considerably across diverse pathophysiological contexts. Experimental data suggest that these protective effects are most pronounced in populations with severe baseline microcirculatory impairment, such as those with diabetes, obstructive sleep apnea (OSA), or HIV infection. In contrast, for patients with high dietary sodium intake, those in the early stages of disease, or specific cohorts of women with non-obstructive coronary artery disease, the efficacy of MRAs may be attenuated or statistically insignificant.

This heterogeneity suggests that the clinical utility of MRAs is intrinsically linked to the severity of baseline vascular damage and individual patient characteristics. Consequently, large-scale prospective clinical trials utilizing advanced myocardial perfusion imaging are warranted to further investigate these trends, with a specific focus on novel non-steroidal MRAs like finerenone. Such research is essential to refine clinical application strategies and to fully elucidate the pharmacological mechanisms by which MRAs modulate the coronary microcirculation across diverse patient populations.
